# Supplementary material for: Developing better digital health measures of Parkinson’s disease using free living data and a crowdsourced data analysis challenge
Source: PLOS Digit Health. 2023 Mar 28;2(3):e0000208. doi: 10.1371/journal.pdig.0000208 (PMC10047543; doi:10.1371/journal.pdig.0000208)
Supplement: S1 Table — (PDF) [file pdig.0000208.s001.pdf]

S1 Table: Number of records (sensor data plus paired label) in the training/test splits for the CIS-PD cohort.

	On/off		Dyskinesia		Tremor	
Subject ID	Training	Test	Training	Test	Training	Test
1004	82	27	82	27	82	27
1006	37	12	0	0	37	12
1007	276	91	299	99	299	99
1019	45	15	45	15	45	15
1020	195	65	0	0	195	65
1023	106	35	106	35	106	35
1032	177	59	0	0	177	59
1034	40	14	40	14	40	14
1038	207	70	207	70	207	70
1039	130	43	130	43	0	0
1043	34	12	34	12	34	12
1044	72	24	72	24	0	0
1046	0	0	0	0	67	22
1048	91	30	91	30	91	30
1049	82	27	82	27	82	27
1051	193	63	0	0	0	0
