# Supplementary material for: Developing better digital health measures of Parkinson’s disease using free living data and a crowdsourced data analysis challenge
Source: PLOS Digit Health. 2023 Mar 28;2(3):e0000208. doi: 10.1371/journal.pdig.0000208 (PMC10047543; doi:10.1371/journal.pdig.0000208)
Supplement: S2 Table — (PDF) [file pdig.0000208.s002.pdf]

S2 Table: Number of records (sensor data plus paired label) in the training/test splits for the REAL-PD cohort.

	On/off		Dyskinesia		Tremor	
Subject ID	Training	Test	Training	Test	Training	Test
hbv012	0	0	0	0	42	13
hbv013	65	22	65	22	67	22
hbv014	46	15	0	0	0	0
hbv017	0	0	68	23	0	0
hbv018	0	0	32	10	0	0
hbv022	55	18	0	0	56	18
hbv023	0	0	0	0	46	15
hbv038	44	14	0	0	43	14
hbv043	37	12	37	12	0	0
hbv051	40	13	0	0	0	0
hbv054	0	0	54	19	58	19
hbv077	42	14	0	0	0	0
