# Supplementary material for: Developing better digital health measures of Parkinson’s disease using free living data and a crowdsourced data analysis challenge
Source: PLOS Digit Health. 2023 Mar 28;2(3):e0000208. doi: 10.1371/journal.pdig.0000208 (PMC10047543; doi:10.1371/journal.pdig.0000208)
Supplement: S3 Table — (PDF) [file pdig.0000208.s003.pdf]

S3 Table: P-value for association of top 10 features from team dbmi with label for on/off, dyskinesia and tremor

	**on_off***	**dyskinesia***	**tremor***
**quantile__q_0.7**	0.341	0.76241	0.260796
**number_peaks__n_1**	0.220	0.308912	0.453108
**quantile__q_0.2**	0.4014	0.054511	0.80456
**quantile__q_0.4**	0.574647	0.757697	0.613633
**quantile__q_0.8**	0.830643	0.956247	0.872984
**sum_values**	0.825521	0.853557	0.677784
**quantile__q_0.3**	0.850645	0.167818	0.536281
**fft_coefficient__coeff_0__attr_"real"**	0.884536	0.738591	0.89858
**mean**	0.748474	0.919004	0.636074
**fft_coefficient__coeff_0__attr_"abs"**	0.942655	0.687168	0.575989

* Shown are the p-values for testing for association between magnitude of feature SHAP value and label. For each column, a t-test was performed to compare models trained on the column label against models trained on other labels. No tests were significant at $\alpha = 0.05$ implying that none of the features were specific to individual phenotypes.
