# Supplementary material for: Developing better digital health measures of Parkinson’s disease using free living data and a crowdsourced data analysis challenge
Source: PLOS Digit Health. 2023 Mar 28;2(3):e0000208. doi: 10.1371/journal.pdig.0000208 (PMC10047543; doi:10.1371/journal.pdig.0000208)
Supplement: S4 Table — (PDF) [file pdig.0000208.s004.pdf]

S4 Table: Association (Kendall's tau) of subject characteristics with model improvement (on/off medication)

		dbmi		HaProzdor		ROC BEAT-PD		hecky		Yuanfang Guan		Problem Solver		Meta-Analysis p-val
		tau	p-val	tau	p-val	tau	p-val	tau	p-val	tau	p-val	tau	p-val	
	n	0.225	0.149	0.107	0.495	0.330	0.034	0.172	0.270	0.234	0.134	0.189	0.224	0.158
	Age	0.048	0.804	0.048	0.804	-0.106	0.585	0.144	0.457	-0.144	0.457	0.144	0.457	0.905
UPDRS	Part I	0.250	0.219	0.333	0.102	0.083	0.682	0.270	0.183	0.250	0.219	0.499	0.014	0.131
	Part II	0.217	0.272	0.276	0.162	0.059	0.764	0.177	0.369	0.039	0.842	0.177	0.369	0.399
	Part IV	0.000	1.000	-0.039	0.842	0.020	0.920	-0.099	0.617	0.158	0.424	0.375	0.058	0.710
	Part III (Off)	0.260	0.221	0.416	0.050	0.286	0.178	0.312	0.142	0.338	0.111	0.260	0.221	0.127
	Part III (On)	0.107	0.621	0.267	0.217	0.080	0.711	0.107	0.621	0.053	0.805	-0.107	0.621	0.680
	Reporting Lag	-0.067	0.770	-0.105	0.626	0.067	0.770	-0.048	0.846	0.124	0.559	0.105	0.626	0.946
	Label Variance	0.543	0.004	0.429	0.027	0.486	0.011	0.448	0.021	0.543	0.004	0.410	0.036	0.010
