# Supplementary material for: Developing better digital health measures of Parkinson’s disease using free living data and a crowdsourced data analysis challenge
Source: PLOS Digit Health. 2023 Mar 28;2(3):e0000208. doi: 10.1371/journal.pdig.0000208 (PMC10047543; doi:10.1371/journal.pdig.0000208)
Supplement: S5 Table — (PDF) [file pdig.0000208.s005.pdf]

S5 Table: Association (Kendall's tau) of subject characteristics with model improvement (dyskinesia)

		ROC BEAT-PD		Yuanfang Guan		hecky		Meta-Analysis p-val
		**tau**	**p-val**	**tau**	**p-val**	**tau**	**p-val**	
	n	-0.202	0.279	0.034	0.857	-0.134	0.470	0.692
	Age	-0.537	0.023	-0.278	0.240	-0.241	0.309	0.273
UPDRS	Part I	-0.101	0.681	0.060	0.805	-0.141	0.565	0.856
	Part II	-0.305	0.205	-0.229	0.342	-0.076	0.751	0.529
	Part IV	-0.057	0.813	-0.094	0.694	-0.623	0.009	0.417
	Part III (Off)	0.386	0.125	0.114	0.652	0.205	0.417	0.496
	Part III (On)	0.092	0.717	0.138	0.587	0.414	0.103	0.533
	Reporting Lag	-0.309	0.218	-0.418	0.087	-0.455	0.060	0.220
	Label Variance	0.636	0.006	0.382	0.121	0.127	0.648	0.231
