# Supplementary material for: Developing better digital health measures of Parkinson’s disease using free living data and a crowdsourced data analysis challenge
Source: PLOS Digit Health. 2023 Mar 28;2(3):e0000208. doi: 10.1371/journal.pdig.0000208 (PMC10047543; doi:10.1371/journal.pdig.0000208)
Supplement: S6 Table — (PDF) [file pdig.0000208.s006.pdf]

S6 Table: Association (Kendall's tau) of subject characteristics with model improvement (tremor)

		Yuanfang Guan		dbmi		ROC BEAT-PD		HaProzdor		Problem Solver		hecky		Meta-Analysis p-val
		**tau**	**p-val**	**tau**	**p-val**	**tau**	**p-val**	**tau**	**p-val**	**tau**	**p-val**	**tau**	**p-val**	
	n	-0.131	0.440	-0.190	0.261	0.083	0.623	0.000	1.000	0.202	0.233	-0.024	0.888	0.951
	Age	-0.194	0.359	0.116	0.582	-0.090	0.669	-0.260	0.221	-0.297	0.160	-0.271	0.199	0.417
UPDRS	Part I	0.406	0.062	0.352	0.106	0.406	0.062	0.123	0.575	0.433	0.046	0.379	0.081	0.086
	Part II	0.278	0.196	0.464	0.031	0.066	0.758	0.133	0.537	0.066	0.758	0.013	0.951	0.404
	Part IV	0.225	0.295	-0.066	0.758	0.331	0.123	-0.187	0.387	0.331	0.123	-0.013	0.951	0.611
	Part III (Off)	0.330	0.160	0.257	0.274	0.183	0.435	0.537	0.023	0.294	0.212	0.330	0.160	0.157
	Part III (On)	-0.075	0.753	0.112	0.637	0.000	1.000	0.321	0.180	-0.112	0.637	0.037	0.875	0.836
	Reporting Lag	0.179	0.435	0.179	0.435	0.385	0.076	-0.039	0.855	0.282	0.204	-0.154	0.510	0.699
	Label Variance	0.590	0.004	0.282	0.204	0.282	0.204	0.090	0.669	0.385	0.076	0.205	0.367	0.132
