# Supplementary material for: Developing better digital health measures of Parkinson’s disease using free living data and a crowdsourced data analysis challenge
Source: PLOS Digit Health. 2023 Mar 28;2(3):e0000208. doi: 10.1371/journal.pdig.0000208 (PMC10047543; doi:10.1371/journal.pdig.0000208)
Supplement: S7 Table — (PDF) [file pdig.0000208.s007.pdf]

S7 Table: Validation of models in clinically labeled segments (on/off medication)

	dbmi		haProzdor		ROC BEAT-PD		Yuanfang Guan	
**Subject ID**	**Correlation**	**P-value***	**Correlation**	**P-value**	**Correlation**	**P-value**	**Correlation**	**P-value**
1004	0.324	0.002	0.387	1.41E-04	0.316	0.002	0.493	4.76E-06
1007	0.176	0.111	0.109	0.212	0.046	0.369	-0.093	0.742
1019	0.036	0.365	-0.015	0.557	0.335	3.69E-04	-0.166	0.943
1020	-0.006	0.508	0.271	0.174	0.296	0.152	-0.028	0.537
1023	-0.051	0.689	-0.063	0.733	0.033	0.372	0.207	0.021
1032	0.348	2.59E-04	-0.040	0.650	-0.292	0.998	0.040	0.349
1038	0.146	0.092	0.225	0.019	-0.187	0.957	0.495	9.80E-07
1039	0.275	0.004	-0.002	0.507	0.045	0.331	0.027	0.397
1043	0.377	6.58E-05	0.268	0.004	0.144	0.079	0.336	4.96-04
1044	0.361	1.64E-04	NA**	NA	-0.188	0.968	0.092	0.188
1046	NA	NA	NA	NA	0.152	0.067	NA	NA
1048	0.258	0.011	0.372	3.11E-04	-0.109	0.833	0.274	0.008
1049	-0.192	0.960	-0.032	0.612	-0.186	0.955	0.017	0.439
1051	0.280	0.003	-0.033	0.628	0.410	1.40E-05	-0.367	1.000
**Meta-Analysis*** **		**1.80e-12**		**1.04e-05**		**9.81e-06**		**6.28E-10**

* One-sided *p*-value
** NA indicates that a model could not produce a prediction for this individual or that the prediction was constant for all segments
*** Unadjusted *p*-value
