# Supplementary material for: Developing better digital health measures of Parkinson’s disease using free living data and a crowdsourced data analysis challenge
Source: PLOS Digit Health. 2023 Mar 28;2(3):e0000208. doi: 10.1371/journal.pdig.0000208 (PMC10047543; doi:10.1371/journal.pdig.0000208)
Supplement: S8 Table — (PDF) [file pdig.0000208.s008.pdf]

S8 Table: Validation of models in clinically labeled segments (dyskinesia)

	ROC BEAT-PD		Yuanfang Guan		HaProzdor		dbmi	
**Subject ID**	**Correlation**	**P-value***	**Correlation**	**P-value**	**Correlation**	**P-value**	**Correlation**	**P-value**
1004	-0.231	0.986	-0.371	1.000	-0.131	0.890	-0.306	0.998
1020	-0.299	0.861	NA*	NA	NA	NA	NA	NA
1023	0.107	0.138	-0.034	0.634	NA	NA	0.075	0.224
1039	0.115	0.121	0.015	0.438	0.047	0.318	-0.011	0.541
1043	0.068	0.245	-0.229	0.990	NA	NA	0.051	0.301
1048	0.286	0.003	0.118	0.136	NA	NA	0.290	0.004
1049	0.049	0.321	-0.076	0.762	-0.155	0.929	-0.004	0.513
**Meta-Analysis*** **		**0.035**		**0.850**		**0.849**		**0.082**

* One-sided *p*-values reported
** NA indicates that a model could not produce a prediction for this individual or that the prediction was constant for all segments
*** Unadjusted *p*-value
