# Supplementary material for: Developing better digital health measures of Parkinson’s disease using free living data and a crowdsourced data analysis challenge
Source: PLOS Digit Health. 2023 Mar 28;2(3):e0000208. doi: 10.1371/journal.pdig.0000208 (PMC10047543; doi:10.1371/journal.pdig.0000208)
Supplement: S9 Table — (PDF) [file pdig.0000208.s009.pdf]

S9 Table: Validation of models in clinically labeled segments (tremor)

	Yuanfang Guan		dbmi		ROC BEAT-PD		haProzdor	
**Subject ID**	**Correlation**	**P-value***	**Correlation**	**P-value**	**Correlation**	**P-value**	**Correlation**	**P-value**
1004	0.145	0.086	0.215	0.024	0.032	0.384	0.480	8.38E-07
1007	-0.038	0.614	0.025	0.428	-0.043	0.627	NA	NA
1019	-0.050	0.694	-0.078	0.780	-0.146	0.931	-0.059	0.724
1020	0.159	0.286	0.002	0.497	0.168	0.275	0.171	0.271
1023	-0.049	0.690	-0.040	0.658	-0.010	0.539	NA	NA
1032	-0.050	0.696	0.392	1.95E-05	0.100	0.155	0.037	0.353
1038	0.081	0.222	0.178	0.045	0.040	0.352	0.018	0.432
1039	0.009	0.462	NA	NA	0.122	0.108	NA	NA
1043	0.124	0.104	0.256	0.004	0.126	0.101	0.200	0.021
1046	0.267	0.003	-0.305	0.999	0.077	0.216	NA	NA
1048	0.099	0.179	0.509	3.24E-07	-0.183	0.956	-0.110	0.846
1049	0.096	0.183	0.273	0.004	-0.172	0.949	0.043	0.341
1051	-0.027	0.606	NA	NA	0.093	0.173	NA	NA
**Meta-Analysis*** **		**0.047**		**1.97e-10**		**0.337**		**1.27e-04**

* One-sided *p*-value
** NA indicates that a model could not produce a prediction for this individual or that the prediction was constant for all segments
*** Unadjusted *p*-value
