# Supplementary material for: Developing better digital health measures of Parkinson’s disease using free living data and a crowdsourced data analysis challenge
Source: PLOS Digit Health. 2023 Mar 28;2(3):e0000208. doi: 10.1371/journal.pdig.0000208 (PMC10047543; doi:10.1371/journal.pdig.0000208)
Supplement: S10 Table — (PDF) [file pdig.0000208.s010.pdf]

**S10 Table**: REAL-PD proportion of records with sensor data from the smartphone, the smartwatch or both.

| Subject ID | Smartphone Only | Smartwatch Only | Both |
|---|---|---|---|
| hbv012 | 0.38 | 0.02 | 0.60 |
| hbv013 | 0.15 | 0.36 | 0.48 |
| hbv014 | 0.00 | 0.02 | 0.98 |
| hbv017 | 0.02 | 0.05 | 0.92 |
| hbv018 | 0.11 | 0.35 | 0.54 |
| hbv022 | 0.04 | 0.09 | 0.87 |
| hbv023 | 0.00 | 0.13 | 0.87 |
| hbv038 | 0.33 | 0.03 | 0.64 |
| hbv043 | 0.02 | 0.16 | 0.82 |
| hbv051 | 0.07 | 0.00 | 0.93 |
| hbv054 | 0.00 | 0.05 | 0.95 |
| hbv077 | 0.10 | 0.03 | 0.86 |
