# Supplementary material for: Developing better digital health measures of Parkinson’s disease using free living data and a crowdsourced data analysis challenge
Source: PLOS Digit Health. 2023 Mar 28;2(3):e0000208. doi: 10.1371/journal.pdig.0000208 (PMC10047543; doi:10.1371/journal.pdig.0000208)
Supplement: S11 Table — (PDF) [file pdig.0000208.s011.pdf]

S11 Table: REAL-PD medication status harmonization

Original category	On/off	Dyskinesia
Off	1	0
On without dyskinesia	0	0
On with non-troublesome dyskinesia	0	1
On with severe dyskinesia	0	2
