# Supplementary material for: Developing better digital health measures of Parkinson’s disease using free living data and a crowdsourced data analysis challenge
Source: PLOS Digit Health. 2023 Mar 28;2(3):e0000208. doi: 10.1371/journal.pdig.0000208 (PMC10047543; doi:10.1371/journal.pdig.0000208)
Supplement: S1 Fig — Bootstraps (n = 1000) of submissions for (A) SC1: on/off, (B) SC2: dyskinesia, and (C) SC3: tremor. Team models (black) and their ensembles (blue) are ordered by rank. Boxes correspond to the 25th, 50th, and 75th percentiles, and individual points are displayed beyond 1.5*IQR (interquartile range) from the edge of the box. For each sub-challenge, a null model (shown in red) estimated as the subject-specific mean of the training labels was used as a benchmark. (PDF) [file pdig.0000208.s012.pdf]

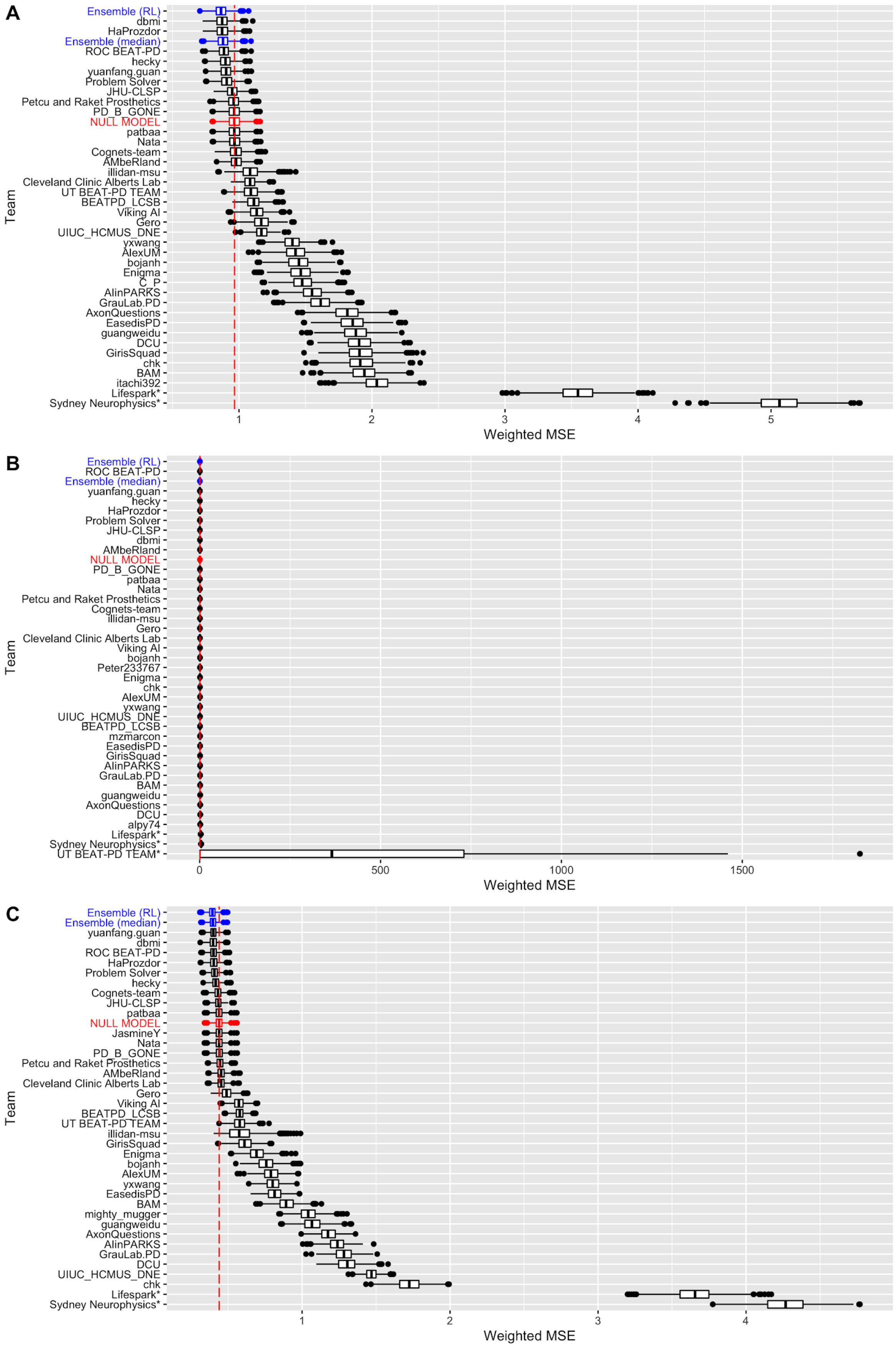
A
Ensemble (RL)
dbmi
HaProzdor
Ensemble (median)
ROC BEAT-PD
hecky
yuanfang.guan
Problem Solver
JHU-CLSP
Petcu and Raket Prosthetics
PD_B_GONE
NULL MODEL
patbaa
Nata
Cognets-team
AMbeRland
illidan-msu
Cleveland Clinic Alberts Lab
UT BEAT-PD TEAM
BEATPD_LCSB
Viking AI
Gero
UIUC_HCMUS_DNE
yxwang
AlexUM
bojanh
Enigma
C_P
AlinPARKS
GrauLab.PD
AxonQuestions
EasedisPD
guangweidu
DCU
GirisSquad
chk
BAM
itachi392
Lifespark*
Sydney Neurophysics*
Team
1
2
3
4
5
Weighted MSE
B
Ensemble (RL)
ROC BEAT-PD
Ensemble (median)
yuanfang.guan
hecky
HaProzdor
Problem Solver
JHU-CLSP
dbmi
AMbeRland
NULL MODEL
PD_B_GONE
patbaa
Nata
Petcu and Raket Prosthetics
Cognets-team
illidan-msu
Gero
Cleveland Clinic Alberts Lab
Viking AI
bojanh
Peter233767
Enigma
chk
AlexUM
yxwang
UIUC_HCMUS_DNE
BEATPD_LCSB
mzmarcon
EasedisPD
GirisSquad
AlinPARKS
GrauLab.PD
BAM
guangweidu
AxonQuestions
DCU
alpy74
Lifespark*
Sydney Neurophysics*
UT BEAT-PD TEAM*
Team
0
500
1000
1500
Weighted MSE
C
Ensemble (RL)
Ensemble (median)
yuanfang.guan
dbmi
ROC BEAT-PD
HaProzdor
Problem Solver
hecky
Cognets-team
JHU-CLSP
patbaa
NULL MODEL
JasmineY
Nata
PD_B_GONE
Petcu and Raket Prosthetics
AMbeRland
Cleveland Clinic Alberts Lab
Gero
Viking AI
BEATPD_LCSB
UT BEAT-PD TEAM
illidan-msu
GirisSquad
Enigma
bojanh
AlexUM
yxwang
EasedisPD
BAM
mighty_mugger
guangweidu
AxonQuestions
AlinPARKS
GrauLab.PD
DCU
UIUC_HCMUS_DNE
chk
Lifespark*
Sydney Neurophysics*
Team
1
2
3
4
Weighted MSE

S1 Fig: Bootstraps (n = 1000) of submissions for (A) SC1: on/off, (B) SC2: dyskinesia, and (C) SC3: tremor. Team models (black) and their ensembles (blue) are ordered by rank. Boxes correspond to the 25th, 50th, and 75th percentiles, and individual points are displayed beyond 1.5*IQR (interquartile range) from the edge of the box. For each sub-challenge, a null model (shown in red) estimated as the subject-specific mean of the training labels was used as a benchmark.
