# Supplementary material for: Developing better digital health measures of Parkinson’s disease using free living data and a crowdsourced data analysis challenge
Source: PLOS Digit Health. 2023 Mar 28;2(3):e0000208. doi: 10.1371/journal.pdig.0000208 (PMC10047543; doi:10.1371/journal.pdig.0000208)
Supplement: S2 Fig — Interaction effects outweigh main effects in all models. (PDF) [file pdig.0000208.s013.pdf]

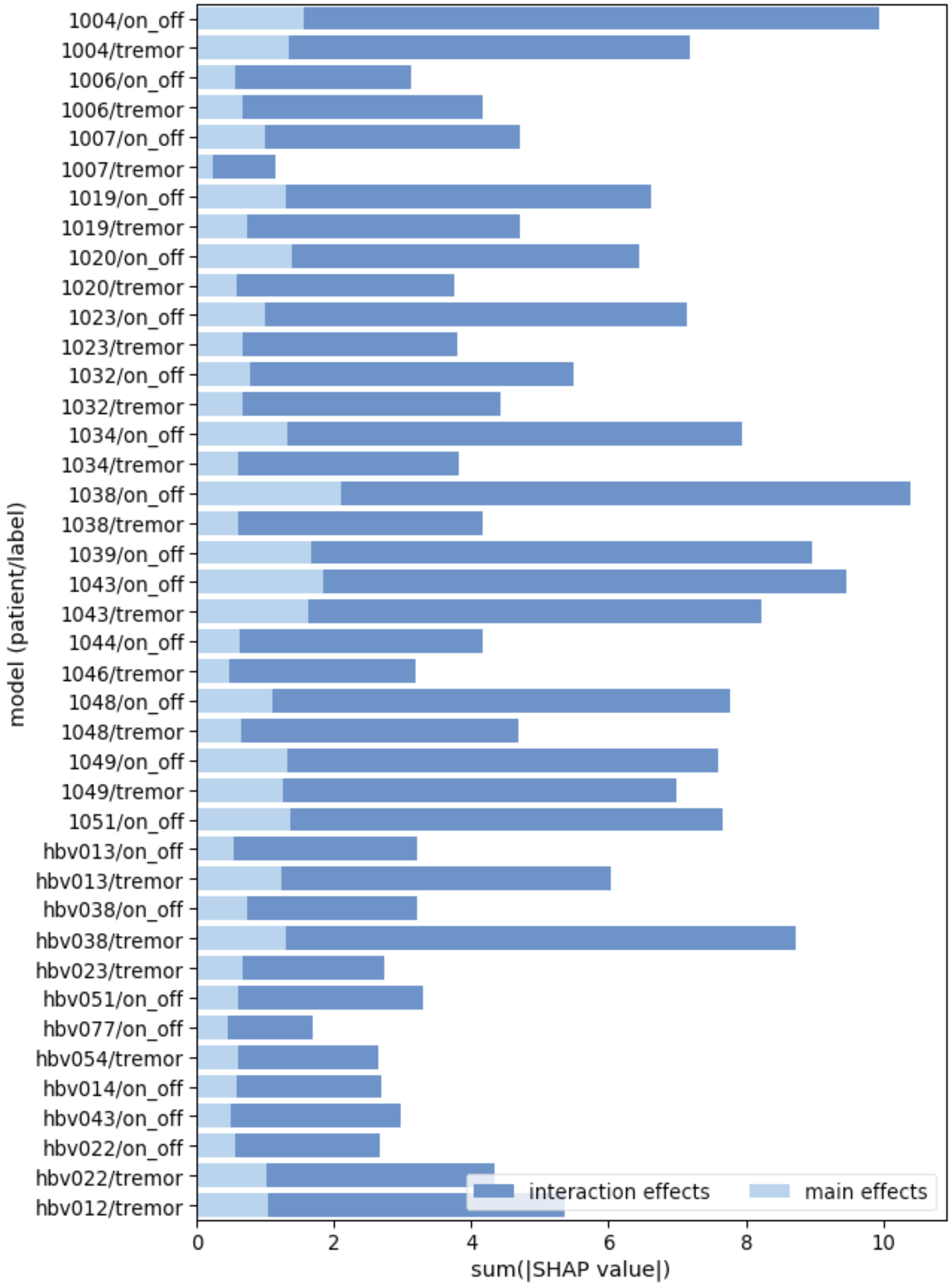

S2 Fig: Sum of magnitudes of SHAP interaction values with main and interaction effects separately, shown for the winning models of team dbmi in SC1 and SC3. Interaction effects outweigh main effects in all models
