# Supplementary material for: Developing better digital health measures of Parkinson’s disease using free living data and a crowdsourced data analysis challenge
Source: PLOS Digit Health. 2023 Mar 28;2(3):e0000208. doi: 10.1371/journal.pdig.0000208 (PMC10047543; doi:10.1371/journal.pdig.0000208)
Supplement: S3 Fig — quantile__q_{0.2, 0.3, 0.4, 0.7, 0.8} are the 20, 30, 40, 70, and 80th data percentiles, respectively; number_peaks__n_1 is defined as the number of peaks of at least support 1; sum_values is the sum over time series values; mean is the mean time series value; and fft_coefficient__coeff_0__attr_{”real”, “abs”} are the real component and absolute value of the 0th coefficient of the fast Fourier Transform (0 Hz), respectively. (PDF) [file pdig.0000208.s014.pdf]

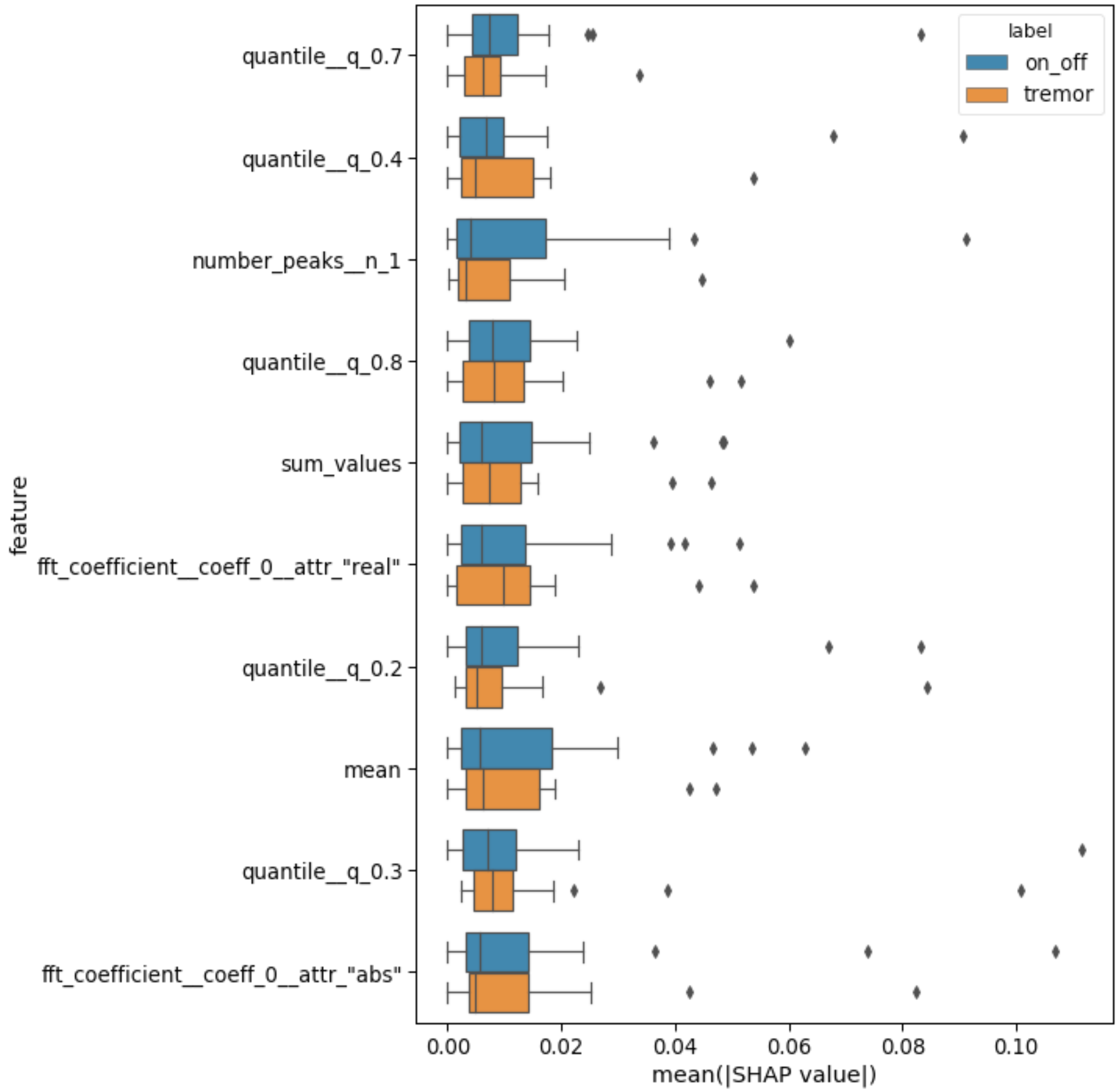

S3 Fig: Distributions of SHAP magnitude for the top 10 features over models for *tremor* (SC3) and *on_off* (SC1) labels for the winning models of team dbmi. *quantile__q_{0.2, 0.3, 0.4, 0.7, 0.8}* are the 20, 30, 40, 70, and 80th data percentiles, respectively; *number_peaks__n_1* is defined as the number of peaks of at least support 1; *sum_values* is the sum over time series values; *mean* is the mean time series value; and *fft_coefficient__coeff_0__attr_{"real", "abs"}* are the real component and absolute value of the 0th coefficient of the fast Fourier Transform (0 Hz), respectively.
