# Supplementary material for: Developing better digital health measures of Parkinson’s disease using free living data and a crowdsourced data analysis challenge
Source: PLOS Digit Health. 2023 Mar 28;2(3):e0000208. doi: 10.1371/journal.pdig.0000208 (PMC10047543; doi:10.1371/journal.pdig.0000208)
Supplement: S4 Fig — Some features are definitionally equivalent (eg 0 Hz FFT component and mean), while others are very similar in definition (eg 30th and 40th percentiles) contributing to strong correlation. (PDF) [file pdig.0000208.s015.pdf]

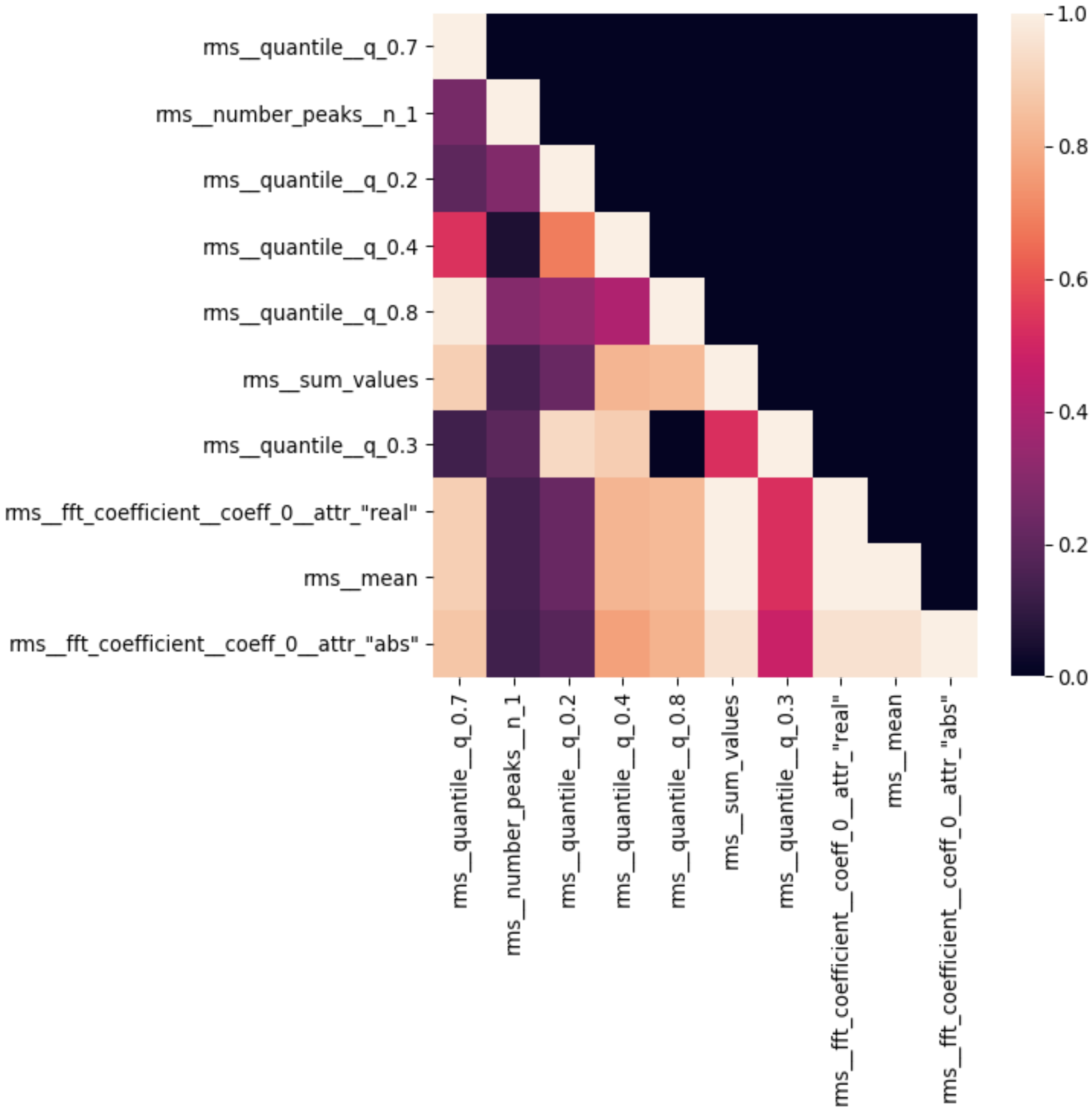

S4 Fig: Heatmap indicating strong correlation among top 10 features from the winning models of team dbmi in SC1 and SC3. Some features are definitionally equivalent (eg 0 Hz FFT component and mean), while others are very similar in definition (eg 30th and 40th percentiles) contributing to strong correlation.
