# Supplementary material for: Developing better digital health measures of Parkinson’s disease using free living data and a crowdsourced data analysis challenge
Source: PLOS Digit Health. 2023 Mar 28;2(3):e0000208. doi: 10.1371/journal.pdig.0000208 (PMC10047543; doi:10.1371/journal.pdig.0000208)
Supplement: S5 Fig — Also shown are the median scores of each category, as well as those of the five base predictors generated by the individual teams’ methods. (PDF) [file pdig.0000208.s016.pdf]

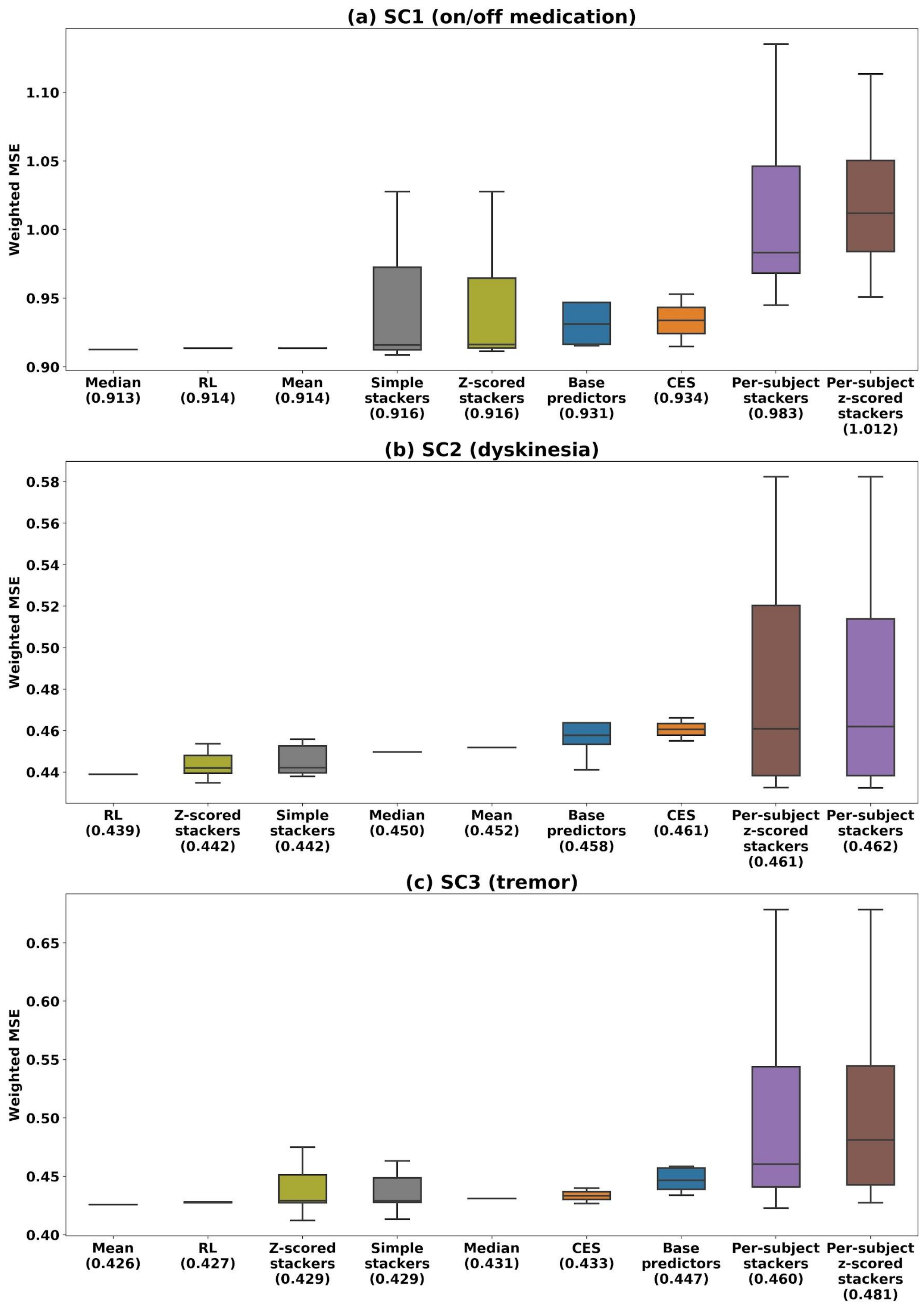

S5 Fig: Distributions of performance of the various categories of ensembles constructed for SC1-3 on the corresponding validation (6th) fold of the corresponding training sets. Also shown are the median scores of each category, as well as those of the five base predictors generated by the individual teams' methods.
