# Supplementary material for: Developing better digital health measures of Parkinson’s disease using free living data and a crowdsourced data analysis challenge
Source: PLOS Digit Health. 2023 Mar 28;2(3):e0000208. doi: 10.1371/journal.pdig.0000208 (PMC10047543; doi:10.1371/journal.pdig.0000208)
Supplement: S8 Fig — (A) Weighted by sqrt(n) and (B) as a percentage of the null model MSE. Teams are ordered by overall rank. (PDF) [file pdig.0000208.s019.pdf]

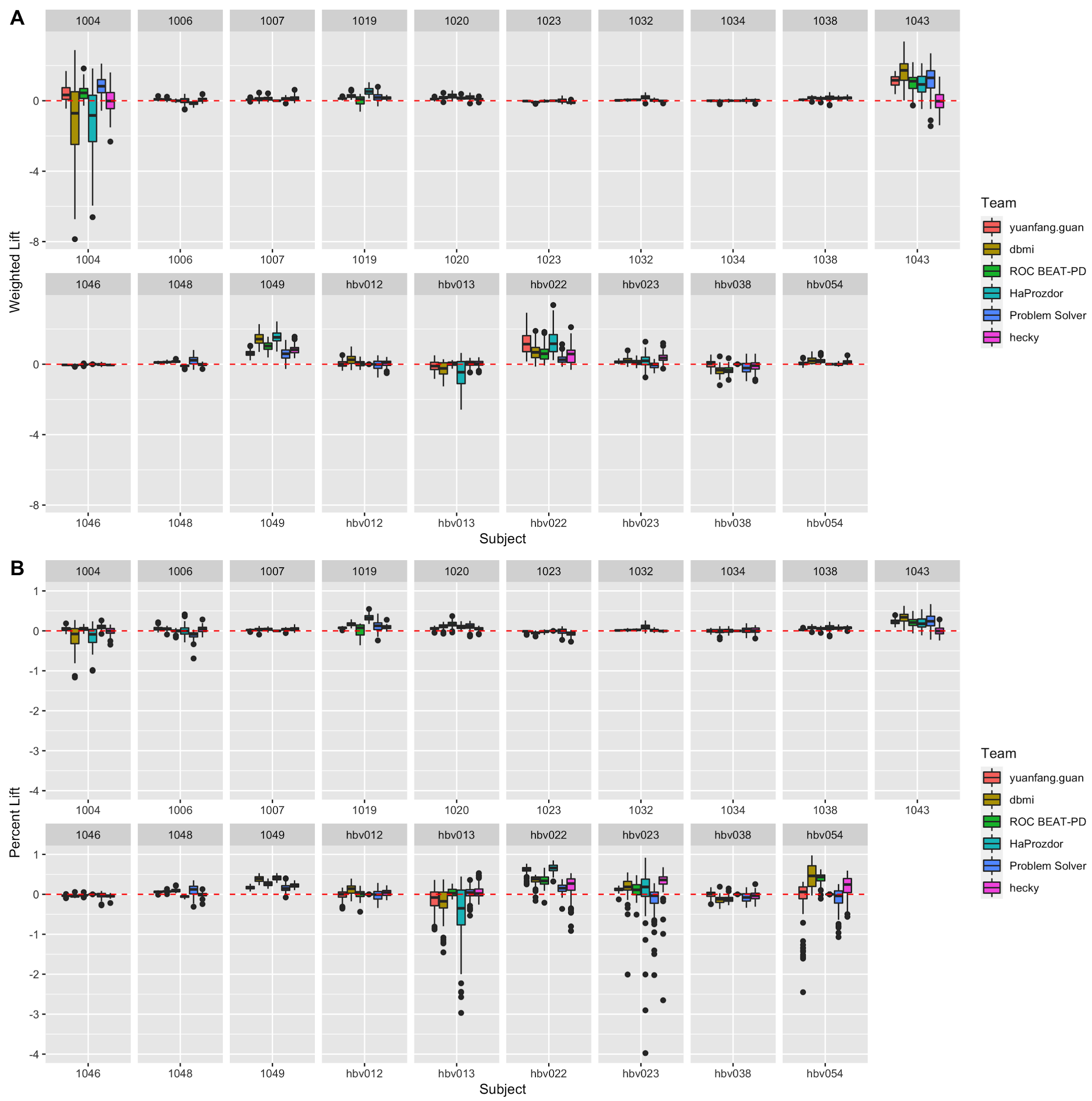

S8 Fig: Tremor subject-specific lift. (A) Weighted by sqrt(n) and (B) as a percentage of the null model MSE. Teams are ordered by overall rank.
