# Supplementary figures and images for: Developing better digital health measures of Parkinson’s disease using free living data and a crowdsourced data analysis challenge
Source: PLOS Digit Health. 2023 Mar 28;2(3):e0000208. doi: 10.1371/journal.pdig.0000208 (PMC10047543; doi:10.1371/journal.pdig.0000208)

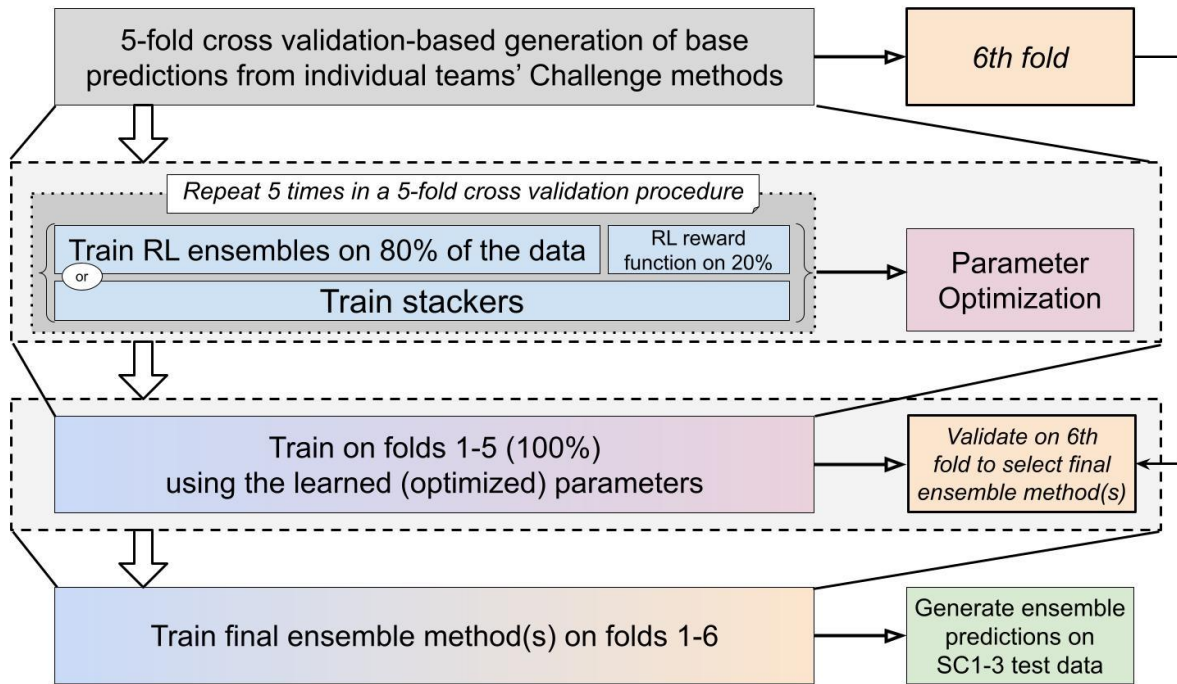

**S9 Fig:** Data-driven process used to train and evaluate heterogeneous ensembles for SC1-3.

Supplement: S9 Fig — (PDF) [file pdig.0000208.s020.pdf]
