# Supplementary material for: Developing better digital health measures of Parkinson’s disease using free living data and a crowdsourced data analysis challenge
Source: PLOS Digit Health. 2023 Mar 28;2(3):e0000208. doi: 10.1371/journal.pdig.0000208 (PMC10047543; doi:10.1371/journal.pdig.0000208)
Supplement: S1 Acknowledgements — (PDF) [file pdig.0000208.s022.pdf]

Supplemental Acknowledgements

The BEAT-PD DREAM Challenge Consortium

Anastasia Adriano[19], Varun Aggarwal[20], Jay Alberts[21], Izhar Bar-Gad[5], Brett Beaulieu-Jones[4,8], Maedeh Beheshti[20], Saurabhchand Bhati[22], Henryk Borzymowski[2], Alma Cantu[23], Nanxin Chen[22], Silvia Del Din[24], Amey Desai [25], Yuval El-Hanani[5], Luc J.W. Evers[13,14], Luca Foschini[15], Nilav Ghosh[26], Marie-Philippe Gill[22], Enrico Glaab[27], Jann Goschenhofer[2],Yuanfang Guan[3], Jonathan Hampton[28], Yidi Huang[4], Monica Javidnia[6,9], Arun Jayaraman[12], Bruno Jedynak[19], Sonal Joshi[22], Mark S. Keller[4], Sheraz Khan[29], Jeff Knisley[28], Abhiroop Kumar[26], Christoph Kurz[30], Yan-chak Li[10], Alex Mariakakis[16], Ayala Matzner[5], Lars Lau Raket[31], Encarna Micó Amigo[24], Laureano Moro-Velaquez[22], Adonay S. Nunes[32], Larsson Omberg[1], Alex Page[6,7], Gaurav Pandey[10], Jiajie Peng[33], Jagdeep T. Podichetty[20], Alisa Prusokiene[35], Augustinas Prusokas[34], Rana Zia Ur Rehman[24], Renata Retkute[36], Sakshi Sardar[20], Mohammed Saqib[4],Nicholas Shawen[17,18], Solveig K. Sieberts[1], Greta Smith[6,9], Phil Synder[1], Ana Stanescu[11], Charles S. Venuto[6,9], Yuxian Wang[33], Robert Zielinski[6,9],

[1] Sage Bionetworks, Seattle, Washington, United States of America
[2] Independent researcher
[3] Department of Computational Medicine and Bioinformatics, University of Michigan, Ann Arbor, Michigan, United States of America
[4] Department of Biomedical Informatics, Harvard Medical School, Boston, Massachusetts, United States of America
[5] Gonda Brain Research Center, Bar Ilan University, Ramat Gan, Israel
[6] Center for Health + Technology, University of Rochester Medical Center, Rochester, New York, United States of America
[7] Cardiology Division, University of Rochester Medical Center, Rochester, New York, United States of America
[8] Department of Neurology, Brigham and Women's Hospital, Boston, Massachusetts, United States of America
[9] Department of Neurology, University of Rochester, Rochester, New York, United States of America
[10] Department of Genetics and Genomic Sciences, Icahn School of Medicine at Mount Sinai, New York, New York, United States of America
[11] Department of Computing and Mathematics, University of West Georgia, Carrollton, Georgia, United States of America
[12] Max Nader Center for Rehabilitation Technologies & Outcomes Research, Shirley Ryan AbilityLab, Chicago, Illinois, United States of America
[13] Donders Institute for Brain, Cognition and Behaviour, Department of Neurology, Radboud University Medical Center, Nijmegen, the Netherlands
[14] Institute for Computing and Information Sciences, Radboud University, Nijmegen, the Netherlands

[15] Evidation Health, Santa Barbara, California, United States of America
[16] Department of Computer Science, University of Toronto, Toronto, Ontario, Canada
[17] Max Nader Research Technologies & Outcomes Lab, Shirley Ryan AbilityLab, Chicago, Illinois, United States of America
[18] Medical Scientist Training Program, Northwestern University Feinberg School of Medicine, Chicago, Illinois, United States of America
[19] Fariborz Maseeh Department of Mathematics + Statistics, Portland State University, Portland, Oregon, United States of America
[20] Critical Path Institute, Tucson, Arizona, United States of America
[21] Department of Biomedical Engineering, Cleveland Clinic, Cleveland, Ohio, United States of America
[22] Department of Electrical and Computer Engineering, Johns Hopkins University, Baltimore, Maryland, United States of America
[23] School of Computing, Newcastle University, Newcastle upon Tyne, United Kingdom
[24] Translational and Clinical Research Institute, Newcastle University, Newcastle upon Tyne, United Kingdom
[25] Lifespark Technologies, Bombay, Mumbai, India
[26] Optum Global Solutions, Hyderabad, India
[27] Luxembourg Centre for Systems Biomedicine, University of Luxembourg, Esch-sur-Alzette, Luxembourg
[28] Department of Mathematics and Statistics, East Tennessee State University, Johnson City, Tennessee, United States of America
[29] Department of Radiology, Massachusetts General Hospital, Harvard Medical School, Boston, Massachusetts, United States of America
[30] Helmholtz Zentrum München, Institute of Health Economics and Health Care Management, Neuherberg, Germany
[31] Department of Data Science, H. Lundbeck A/S, Valby, Denmark
[32] Department of Neurology, Massachusetts General Hospital, Harvard Medical School, Boston, Massachusetts, United States of America
[33] School of Computer Science, Northwestern Polytechnical University, Xi’an, China
[34] Department of Life Sciences, Imperial College London, United Kingdom
[35] School of Natural and Environmental Sciences, Newcastle University, United Kingdom
[36] Department of Plant Sciences, University of Cambridge, United Kingdom
