# Supplementary material for: Developing better digital health measures of Parkinson’s disease using free living data and a crowdsourced data analysis challenge
Source: PLOS Digit Health. 2023 Mar 28;2(3):e0000208. doi: 10.1371/journal.pdig.0000208 (PMC10047543; doi:10.1371/journal.pdig.0000208)
Supplement: Attachment — Submitted filename: BEAT-PD Response to Reviewers2.pdf [file pdig.0000208.s023.pdf]

In addition to the point-by-point response to reviewers below, we note that the changes requested to our Financial Disclosure and Competing Interests statements did not appear in the PDF of our submission. As such, we are including them here to ensure they are seen by the editorial team.

Financial Disclosures:
The BEAT-PD Challenge was funded by the Michael J. Fox Foundation (MJFF) in a grant to LO. MJFF played an advisory role in the design of the challenge, but played no role in the execution, analysis or adjudication, nor in the decision to publish or preparation of the manuscript.

The salaries of SKS, AJ, AM2, NS, PS and LO were partially supported by funds from MJFF.

The ensemble computations were partly enabled by Scientific Computing resources at the Icahn School of Medicine at Mount Sinai, and YL and GP were supported by NIH R01HG011407-01A1.

YG is funded separately by the Michael J. Fox Foundation. BKBJ was supported by a grant from NIH NINDS (award #: K99NS114850). MSK is supported by a grant from NIH (award #: 5T32HG002295-18).
CSV, GS, and RZ received research support funded by the NIH NINDS under award number P50NS108676.

Competing Interests:

I have read the journal's policy and the authors of this manuscript have the following competing interests:

YG serves as scientific advisor for Eli Lilly and Company and Merck & Co.; serves as scientific advisor and receives grants from Merck KGaA. YH has grant funding through BBJ from Sanofi S.A. and UCB for unrelated projects. BBJ has grant funding from Sanofi S.A. and UCB for unrelated projects. AJ is funded by MJ Fox Foundation for data curation.

All other authors report no competing interests.

Response to Reviewers

Reviewer #1: This article presents the findings of the Beat-PD challenge. The purpose of this challenge has been to build a model capable of predict symptom severity for Parkinson’s disease based on a wearable sensor (smartwatch). The challenge consisted in three separate tasks, and around 37-38 teams have participated in each task. The article focuses on

presenting a summary of the approaches taken by the winning teams, and also proposes an ensemble method consisting in combining the predictions of five-best performing teams.

Overall the article is well-written, but I found difficult to follow the section on model interpretation. Maybe a table summarising the main techniques used by the different teams would help with this.   This section seems to give insights into the top models used for task 1 and 2, but not for task 3.

This section covers all 3 sub-challenges based on winning models from two teams dbmi (won *both* SC1 and SC3) and ROC BEAT-PD (won SC2). We have clarified the description to make this more clear.This includes updating the Figure legends referenced within this section. It is important to note that the purpose of this section is not to summarize the techniques used by different teams, but to understand the predictive features in the top models. Notably, comparisons across models are difficult since each team has computed their feature sets differently.

Specific changes:
"Team dmbi (winner of SC1 and co-winner of SC3) and Team ROC BEAT-PD (winner of SC2) both used random forest-based[16] machine learning modeling which alloweds us to explore the model feature importance. Team dbmi trained a random forest model on manually extracted signal features from raw data. Separate models were trained for each patient-phenotype combination. To explore the feature importance within team dbmi's SC1 and SC3 models, we computed SHAP values[17] which quantify the importance of features in a way that is comparable across different models. We computed SHAP values for every prediction and SHAP interaction values for a randomly selected subset of predictions (see Methods). In general, we observed that model predictions were multi-factorial in nature. Effects of individual features were small, and main effects were generally outweighed by interaction effects (Figure S2). However, there was general consistency within the top features, even across the two outcomes examined (on/off (SC1) and tremor (SC3)), with the two models sharing 11 of their top 15 features."

For the ensemble modelling, the reasoning behind the use of five methods is not well explained : why not 2, or 3, or all the models that were statistically better than the Null model?

We have added the following language for clarity:
"These teams were selected based on having submitted models that were significantly better than the Null model (nominal bootstrap p-val < 0.05) for at least one subchallenge. One team that met this bar chose not to join this effort and was not included."

It is also unclear how the train/test partitions were split, if sections were randomly assigned to one of these partitions or the partition was based on some type of temporal aspect: this might have an important impact over the generalisability of the models.

Training/test partitions were split randomly. We have added this information to our methods:

“The training and test data were split randomly for each individual separately, keeping the same within-subject label distributions, in order to facilitate subject-specific modeling.”

As also noted, we had segmented the data into non-overlapping segments, so that temporal ordering could not be reconstructed:

“For each segment, the time series data were reported relative to the start of the segment in order to obscure the relative ordering of the segments. The intervening 10-minutes of sensor recordings between each segment were not provided to participants, to prevent reconstruction of segment ordering.”

Reviewer #2: This paper presents the results of challenges on various topics, all related to Parkinson disease. The paper is very dense and the results of the challenges are very briefly summarized, in order to preserve an acceptable length of the paper. All code and data are available online, hence readers could eventually reproduce the results of the research, while considering the paper to provide only general guidelines with respect to the actual experiments.

The paper is worth publishing especially by light of its results: in reinforces the idea of mining important information on the health status of a person from affordable common devices (such as smartphone or smartwatch).

I only have a remark. The authors repeatedly use the phrase "statistically better", "significantly better" or "significantly improved", "significantly outperformed" without mentioning the test that was employed.

Thank you for pointing out this oversight. We have added the following description in the Results section:

“Bootstrap p-values were computed to compare each submission to the Null model.”

And in the Methods section:

“Submissions were scored using the WMSE described above, and 1,000 bootstraps were performed keeping the same number of within-subject observations for all submissions as well as the Null model. Based on these bootstraps, *p*-values of each submission versus the Null model were computed as the proportion of iterations in which the Null model outperformed the submission. A nominal (unadjusted) 0.05 *p*-value was used to select models significantly better than the Null. Additionally, we deemed the top performing model “distinguishable” from the following model using a nominal 0.05 threshold for the *p*-value of the one-sided Wilcoxon signed-rank test for the bootstrap scores.”

We note that the latter heuristic approach for distinguishing between submissions has been used in previous benchmarking challenges (e.g. https://www.ncbi.nlm.nih.gov/pmc/articles/PMC4996969/).

Finally, throughout the results section we have clarified the use of the bootstrap p-values and confidence level used (changes tracked in the manuscript, but omitted here for space).

SHAP values are mentioned and I think it would be useful to introduce them by a short definition, for readers not familiar with the notion.

To better orient the reader, we have added the following sentence:
To explore the feature importance within team dbmi's SC1 and SC3 models, we computed SHAP values[17], which quantify the importance of features in a way that is comparable across different models.

Reviewer #3: This is an interesting paper attempting to objectively measure Parkinson's disease severity using passive sensor data and making use of a collective effort through a public benchmarking challenge. Enabling objective measures of PD based on passive measurements is important as it does not interfere with activitites of daily living of patients. Few research efforts has been published since it is challenging to make sense of sensor data that could be collected through a routine daily activity (for instance cutting grass with a machine, which could be analyzed by an algorithm as tremor).

The approach taken by the authors is very interesting and at at the same time challenging. Something that I appreciate a lot.

I have the following comments, which hopefully can guid the authors to improve their work:
- Please explain what is the rationale for including CIS-PD and REAL-PD. How do they complement to each other?

Both studies employ similar study designs, albeit with different devices, which made their use together natural. We have added the following phrase to the introduction to help the reader:

"The challenge leveraged two datasets: the Clinicians Input Study (CIS-PD)[9,10] and REAL-PD which is also known as the Parkinson@Home Validation Study[11], <u>which both employed similar approaches pairing smartwatch sensor data with patient-reported symptom severity collected frequently, at-home, over multiple days.</u> In both studies data from smart watches (Apple Watch in CIS-PD and Motorola Watch with an Android phone REAL-PD) were collected from patients as they went through their daily lives. Patients also reported symptom severity at 30-minute increments using digital Hauser diaries over the course of multiple days of these studies[12]. The challenge leveraged 2,476 symptom reports from 16 subjects for CIS-PD and 782 symptom reports from 12 subjects for REAL-PD."

- It is not clear how the Null model has been derived. Please add more details.

The Null model is described in the both the Results and Methods section as follows: "Models were compared to a baseline *Null* model that generated predictions according to the subject-specific mean of the training labels, which is the best prediction in the absence of any sensor data."

- The work presents five models that performed well. When the results and findings are presented it is not clear to which model do they belong. For instance, Figure S4 presents correlations for top 10 features. For which model/team and which challenge?

This Figure is referenced in the discussion of model interpretation for team dbmi's models so refers to that model. We have added reference to the team/model for all figure captions and the table title for the table referenced in this section to better orient the reader.

- In relation to my previous comment, since all the models/teams results are presented as a reader it is difficult to follow the "line of thought". My suggestion is to decide the best model per challenge and then present results in the following sections.

It is not clear based on this comment where the reviewer is having difficulty. However, the Model Interpretation section is already organized in this manner. We have added some additional language in this section to orient the reader, specifically to orient the reader that these results are from team dbmi (SC1 and SC3) and team ROC BEAT-PD (SC2). The remaining sections focus on either ensembling across models or pan-model analyses using models from all teams that were statistically better than the Null model. In the later case, there is value in seeing the commonalities/differences between the models, and for all figures/tables, the models are well labeled.

- When it comes to validation e.g. results presented in tables S8-S10, it is not clear to me why the correlation coefficients for the whole sample are not presented.

The entire sample consists of data from multiple individuals. These individuals can look very different from each other in terms of their own perception of severity and variation. As such the challenge was focused on predicting future severity **within subjects.** This was motivated by models being trained on study subjects' self-ratings, which may show a shift with respect to location (average) relative to an expert clinician. In other words, patients know when they're better vs worse, but not necessarily where they lie on the absolute scale severity, because they only know their own experience.Whole sample correlation is only appropriate for population level models, not subject level models.

Furthermore, It is also valuable to see that there are some subjects where the models validate (show significant correlation) and some where they do not. We do provide a cross-subject aggregate summary in the form of a meta-analysis p-value. We have added to the explanation of the approach as follows:

“The top teams were also invited to apply their models to sensor data collected during the completion of short (~30 second) specified tasks for the same study participants in the CIS-PD study. Each of these segments was assessed for symptom severity by a clinical PD expert in order to ascertain the degree to which these models recapitulate clinician-rated severity. Four teams (dbmi, HaProzdor, ROC BEAT-PD, and yuanfang.guan) participated in this exercise and submitted predictions for 1277 segments across 16 subjects. Because of the within-subject nature of this prediction framework and heterogeneity observed above, within-subject correlation between the predicted value and the symptom severity label was used as the measure of accuracy, rather than MSE, in order to account for the fact that patients’ perception of average severity may differ from a physician’s. That is to say, the distributions may be shifted, but we expect the patient- and physician-derived severity ratings to be correlated.”

- The clinical data was used for validation. It is not clear how were they extracted. For instance, how the clinicians (and how many of them?) observed the video recordings? How did they rate and what did they rate?

This information is provided in the methods describing the CIS-PD study. While there are no video recordings (or mentions to that effect) we have clarified that the assessments were done in-person.

“Those participating in a substudy completed at the Northwestern University site[28,29] or identified as having significant motor fluctuations, defined in the study as an average of 2 hours a day in an OFF medication state, also completed additional in-clinic, clinician-rated assessments while wearing the smartwatch. These assessments consisted of a series of functional tasks (e.g. drinking water from a cup, folding towels) performed while a trained clinician rated the presence of tremor and dyskinesia for each limb on 0-4 scales. Assessments were done in-person. The criteria for these scales were based on those used in the MDS-UPDRS Part III (Motor) assessment. An assessment of overall severity of motor symptoms was also made for each task on a similar 0-4 scale (0: Normal, 1: Slight, 2: Mild, 3: Moderate, 4: Severe).”

We have also updated the brief description in the Results section to include the mention of in-person. All descriptions already include mention of “a [trained] clinician” or “a clinical PD expert” with the “a” denoting that it was a single rater.

- Were the challenges different samples?

Tables S1 and S2 outline the samples from each study subject wrt each phenotype. In some instances we have withheld a specific phenotype from a specific study subject because they did not show enough variability to be valuable for the purpose of predictive modeling. This is described in the methods as follows:

“Subjects were filtered on a phenotype-specific basis if they had an insufficient number of observations or label variance. Subjects were included only if they had at least 40 non-missing observations, and at least 2 label categories with 10 or more observations each, or at least 3 label categories with 5 or more observations each.”

Additionally, information from the description of the study design orients the reader that the phenotypes are collected concurrently, We have added language for clarity:

“All participants completed a paper Hauser diary for the 48 hours prior to the first study visit. Participants with motor fluctuations or participating in the substudy, were also asked to complete electronic symptom diaries at half-hour intervals for 48 hours prior to each of the four study visits. Each diary included self reports, on 0-4 scales, of 3 symptoms: whether the participant felt they were in an ON or OFF medication state, as well as the presence of tremor and dyskinesia. Participants received reminders to complete each diary entry through the Fox Wearable Companion app.”

And

“Following the in-home visit, PD patients continued to wear a study-provided smartwatch (Motorola Moto 360 Sport with a custom application collecting raw sensor data) on their most affected side and their own Android smartphone in a pant pocket (as available) for two weeks. During this time, they completed various diaries, including a detailed symptom diary at 30-minute intervals over the course of 2 days. The detailed symptom diary asked patients to rate medication status (OFF, ON without dyskinesia, ON with non-troublesome dyskinesia, ON with severe dyskinesia), as well as tremor severity and slowness of gait on a 1-5 scale at each prompt.”

Finally, we have clarified that the same training/test splits were used across all 3 subchallenges: “Training and test partitions were split within subjects to enable subject-specific models (Tables S1 & S2). The same splits were used across all three subchallenges. Test partition labels were withheld from challenge participants, and they were asked to predict the phenotype severity in the test partition.”

- Have the authors considered to assess responsiveness to treatment changes of the model scores? For instance, between OFF, 30 minutes when receiving dose, and follow-up observations.

While this is an interesting question, it would not be sufficiently powered in our study. PK/PD of levodopa varies based on a number of factors including but not limited to age, time since diagnosis/severity, gender, even stomach contents. Therefore, any such exploration would need to be within-subject. Given that medication intake typically occurs only a few times a day (in contrast to the symptom diaries in these studies, which occur at 30 minute intervals) giving us substantially fewer timepoints to observe and very limited power. Future work wishing to explore these questions might collect medication diaries for weeks to months following an initial study design collecting frequent symptom diaries, with the symptom diaries being used to train the models which are subsequently used to predict symptoms in the more extensive periods with only medication intake.

- Finally, it would be good to add a section on how the authors assessed the validity and reliability of the results they received from the teams?

Thank you for this suggestion. We have added the following paragraph to describe the scoring and comparison to the Null model:

“Participants were provided the test segments without associated labels and submitted predictions for each. Missing values were not allowed. Submissions were scored using the WMSE described above, and 1,000 bootstraps were performed keeping the same number of within-subject observations for all submissions as well as the Null model. Based on these bootstraps, *p*-values of each submission versus the Null model were computed as the proportion of iterations in which the Null model outperformed the submission. A nominal (unadjusted) 0.05 p-value was used to select models significantly better than the Null. Additionally, we deemed the top performing model “distinguishable” from the following model using a nominal 0.05 threshold for the *p*-value of the one-sided Wilcoxon signed-rank test for the bootstrap scores as previously described[30].“
